# A systematic review of randomized controlled trials exploring the effect of immunomodulative interventions on infection, organ failure, and mortality in trauma patients

**DOI:** 10.1186/cc9218

**Published:** 2010-08-05

**Authors:** Nicole E Spruijt, Tjaakje Visser, Luke PH Leenen

**Affiliations:** 1 Department of Surgery, University Medical Centre Utrecht, H.P. G04.228, Heidelberglaan 100, 3584 GX Utrecht, The Netherlands

## Abstract

**Introduction:**

Following trauma, patients may suffer an overwhelming pro-inflammatory response and immune paralysis resulting in infection and multiple organ failure (MOF). Various potentially immunomodulative interventions have been tested. The objective of this study is to systematically review the randomized controlled trials (RCTs) that investigate the effect of potentially immunomodulative interventions in comparison to a placebo or standard therapy on infection, MOF, and mortality in trauma patients.

**Methods:**

A computerized search of MEDLINE, the Cochrane CENTRAL Register of Controlled Trials, and EMBASE yielded 502 studies, of which 18 unique RCTs were deemed relevant for this study. The methodological quality of these RCTs was assessed using a critical appraisal checklist for therapy articles from the Centre for Evidence Based Medicine. The effects of the test interventions on infection, MOF, and mortality rates and inflammatory parameters relative to the controls were recorded.

**Results:**

In most studies, the inflammatory parameters differed significantly between the test and control groups. However, significant changes in infection, MOF, and mortality rates were only measured in studies testing immunoglobulin, IFN-γ, and glucan.

**Conclusions:**

Based on level 1b and 2b studies, administration of immunoglobulin, IFN-γ, or glucan have shown the most promising results to improve the outcome of trauma patients.

## Introduction

Trauma remains the leading cause of death in people under the age of 40 years [1], with multiple organ failure (MOF) accounting for 27.5% of deaths among trauma patients [2]. MOF can be a result of an early over-reaction of the immune system or a late immune paralysis [3]. Several groups have reviewed the changes that occur in the immune system as a result of injury and concluded that pro- and anti-inflammatory reactions play a role in the development of MOF [4-7]. Early MOF, which develops within the first three days after injury without signs of infection, is attributed to an overwhelming leukocyte driven pro-inflammatory response clinically defined as a systemic inflammatory response syndrome (SIRS). Late MOF, on the other hand, is most often associated with infection and occurs more than three days after injury. Late MOF seems to be the result an inadequate specific immune response with diminished antigen presentation, referred to as compensatory anti-inflammatory response syndrome (CARS). Many argue that SIRS and CARS occur simultaneously as a mixed antagonistic response syndrome (MARS) [4,6] and therefore both reactions contribute to the occurrence of infection, sepsis, and MOF.

This knowledge needs to be applied. Which interventions attenuate both the hyper-inflammatory response and immune paralysis and subsequently improve the clinical outcome in trauma patients? Montejo et al. [8] have systematically reviewed the effect of immunonutrition on clinical outcome in trauma patients. Although immunonutrition shortened the time of mechanical ventilation and ICU stay, and resulted in a lower incidence of bacteremias and intra-abdominal infections, the incidence of nosocomial pneumonia, wound infection, urinary tract infection, sepsis, and mortality remain unchanged. Other interventions are needed.

The objective of this paper is to systematically review the randomized controlled trials (RCTs) that investigate the effect of non-nutritional potential immunomodulative interventions in comparison to a placebo or standard therapy on infection, MOF, and mortality in trauma patients.

## Materials and methods

### Search

Studies were found via computerized searches of the MEDLINE and EMBASE databases and the Cochrane CENTRAL Register of Controlled Trials. The search syntax included synonyms of trauma (trauma*, injur*), immunomodulation (immun*, inflammat*), and clinical outcome (infectio*, "organ failure", mortality, surviv*) in the titles, abstracts, and keywords areas. Limits were set to retrieve only studies on humans with high-quality design (meta-analyses, systematic reviews, Cochrane reviews, RCTs, and clinical trials). No limits were imposed on either publication date or language.

### Selection

The search hits were screened for relevance by two authors. Studies were deemed relevant when they investigated the effect of a potentially immunomodulative intervention on clinical outcome in trauma patients. Therefore, studies including patients other than trauma patients (for example, other ICU patients), patients with specific isolated injury (for example, isolated injury to the head or an extremity), or patients with thermal injuries were excluded. Furthermore, patients needed to be randomly allocated to receive a potentially immunomodulative intervention, standard therapy, or a placebo. As the effect of immunonutrition has already been systematically reviewed, studies implementing immunonutrition were excluded. To assess the efficacy of the interventions, only studies reporting clinical outcomes were included. References of the relevant studies were checked for other relevant articles that might have been missed in the computerized search.

### Quality assessment

The methodological quality of each of the studies for which the full text was available was assessed using a checklist for therapy articles from the Centre for Evidence Based Medicine [9,10]. One point was accredited for each positive criterion: the study participants were randomized; the study groups had similar characteristics at baseline; the groups were treated equally except for the test intervention; all patients were accounted for; outcome assessors were blinded to the intervention or used well-defined outcome criteria; and outcomes were compared on an intention-to-treat basis.

### Data abstraction

Data abstraction was completed independently. The studies were searched for patient characteristics (number, age, and injury severity score (ISS)), details of the intervention (test, control, delivery route, and duration) and length of follow-up during which outcome variables were measured. Outcome variables included in the analysis were: infections, overall or specified; MOF or mortality; and inflammatory parameters, cellular or humoral. Definitions of infections given by authors were used, including major and minor infections, pneumonia, sepsis, meningitis, surgical site infections, urinary tract infections, and intra-abdominal abscesses. MOF was defined by MOF scores given by the authors. The efficacy of interventions intended to attenuate the hyper-inflammatory response were compared with those intended to reduce the immune paralysis. Interventions that altered the release of pro-inflammatory cytokines (IL-1β, IL-6, IL-8, TNF-α), active complement factors, leukocyte count, or leukocyte-derived cytotoxic mediators were considered modulators of SIRS. Interventions that altered the release of anti-inflammatory cytokines (IL-10, IL-1RA), antigen-presenting capacity, or bactericidal capacity were considered modulators of CARS.

## Results

### Search and selection

**Figure 1:**
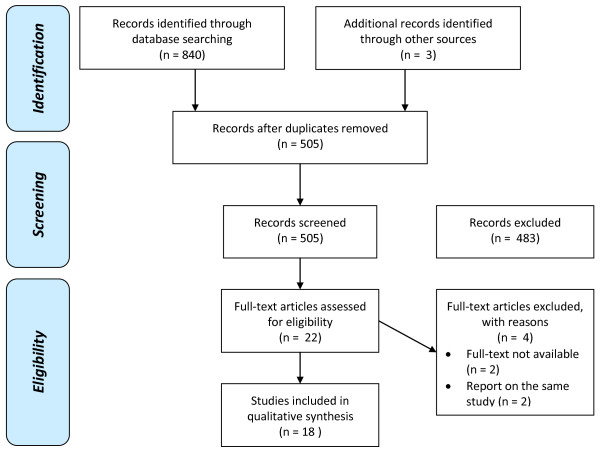
**Study selection**. Computerized search conducted on 4 January, 2010.

After filtering out duplicate studies retrieved from the databases, 502 potentially relevant studies were assessed. Studies were excluded that did not include only trauma patients (444), tested interventions that were not intended to immunomodulate (10), studied the effect of immunonutrition (20), did not report clinical outcome (4), or were non-systematic reviews (5) (Figure 1). The full text was not available for two studies [11,12]. By checking references of the relevant studies, three other relevant studies were found that were missed in the computerized search because the keywords were not included in the titles or abstracts [13-15]. Two articles by Seekamp et al. [16,17] and two articles by Dries et al. [13,18] report on the same study. Therefore, 18 unique RCTs that met the inclusion and exclusion criteria were available for analysis.

### Quality assessment

Using the checklist for therapy articles from the Centre for Evidence Based Medicine [9], all RCTs scored four to six out of a maximum six points (Table 1). Points were lost because the study groups were dissimilar at baseline and/or patients dropped out that were not analyzed on an intention-to-treat basis. Studies scoring a full six points were deemed high-quality RCTs reporting 1b level of evidence [10]. Studies scoring four or five points were deemed of lesser quality and thus reporting 2b level of evidence. Data from all studies were used to determine the effect of potential immunomodulative interventions on clinical outcome in trauma patients.

**Table 1:** Quality assessment

Study	Patients randomized	Groups similar at baseline	Groups treated equally	All patients accounted for	Assessor blinded or objective	Intention to treat analysis	TOTAL (max 6)	Level of Evidence
Browder et al, 1990 [29]	1	1	1	1	1	1	6	1b
Bulger et al, 2008 [19]	1	1	1	1	1	1	6	1b
Croce et al, 1998 [24]	1	0°	1	1	1	1	5	2b
de Felippe et al, 1993 [30]	1	1	1	1	1	0	5	2b
Douzinas et al, 2000 [32]	1	0*	1	1	1	0	4	2b
Dries et al, 1998 [18]	1	1	1	1	1	0	5	2b
Glinz et al, 1985 [20]	1	1	1	1	1	1	6	1b
Livingston et al, 1994 [31]	1	1	1	1	1	1	6	1b
Marzi et al, 1993 [25]	1	1	1	1	1	1	6	1b
Miller & Lim, 1985 [14]	1	n.r.	1	1	1	0	4	2b
Nakos et al, 2002 [26]	1	1	1	1	1	1	6	1b
Nathens et al, 2006 [21]	1	1	1	1	1	1	6	1b
Polk et al, 1992 [22]	1	0°	1	1	1	1	5	2b
Rhee et al, 2000 [23]	1	0	1	1	1	1	5	2b
Rizoli et al, 2006 [27]	1	0	1	1	1	0	4	2b
Seekamp et al, 2004 [16]	1	1	1	1	1	1	6	1b
Vassar et al, 1991 [15]	1	1	1	1	1	1	6	1b
Waydhas et al, 1998 [28]	1	1	1	1	1	0	5	2b

1 = yes; 0 = no; n.r. = not reported, the test group was older; * = the test group had a higher injury severity score, which was corrected for using a multiple regression model.

### Study characteristics

A comparison of the study characteristics of the 18 RCTs reveals marked inter-trial heterogeneity of patients and interventions (Table 2). The number of patients included in the trials ranged from 16 to 268, with five trials studying over 100 patients [19-23]. Of the smaller trials, six were pilot studies [14,24-27]. Three of the trials were phase II trials primarily powered to test dosage and safety, not efficacy [16,23,24]. Patient ages ranged between 13 and 90 years, with the mean age in the 30 s or low 40 s for all studies except those of Rizoli et al. [27] and Seekamp et al. [16,17] in which the mean age was nearer 50 years. Similarly, the ISS ranged from 0 to 75, with the mean ISS in the 20 s or low 30 s for most studies. The studies by Nakos et al. [26] and Waydhas et al. [28] averaged more severely injured patients.

Interventions were intended to attenuate the early overwhelming inflammatory response and diminish the immune paralysis. As many trauma patients are plagued by infections, researchers aimed to augment the host's inflammatory response by stimulating macrophages with glucan [29,30], activating monocytes with dextran [14], upregulating human leukocyte antigen (HLA)-DR expression with interferon (IFN)-γ [18,22,26,31], and providing immunoglobulins [20,32]. As hyper-inflammation causes injury, researchers aimed to taper the host's inflammatory response by infusing leuko-reduced blood [21], prostaglandin E1 [15], antioxidants [25], and antithrombin III [28], which, by blocking thrombin, decreases IL-8 production and sequestration of neutrophils. By blocking a neutrophil receptor that binds to endothelium (CD18) [23] or an adhesion molecule (L-selectin) [16] with an antibody, researchers hoped to prevent neutrophils from extravasating and causing reperfusion injury after hemorrhagic shock. Perflubron is attributed with anti-inflammatory properties because macrophages exposed to it demonstrate significantly less hydrogen peroxide superoxide anion and production [24]. Most of the control groups were given a placebo [15-18,20,22,23,25-32] and four received only standard treatment [14,19,21,24]. The interventions were administered intravenously [14-17,19-21,23,25,27-30,32], subcutaneously [18,22,31], or via inhalation [24,26]. Interventions were initiated as soon as possible after injury by ambulance personnel [19] or as late as 145 hours after hospital admission [30]. The duration of the intervention differed from a single dose to 28 days. The length of follow-up ranged from 10 to 90 days.

**Table 2:** Study characteristics

Study	Patients			Intervention					
			
	n	Age (range)	ISS (range, ± SD)	Test	Control	Delivery	Initiation	Duration	Length of follow-up
Browder et al, 1990 [29]	38	34 (18-65)	24 (8-41)	Glucan	placebo (saline)	i.v.	after exploratory laparotomy or thoracotomy	7 days	10 days
Bulger et al, 2008 [19]	209	38 (13-90)	28 (0-75)	Hypertonic saline + Dextran	Lactated Ringer solution	i.v.	initial reperfusion fluid	single dose	28 days
Croce et al, 1998 [24]	16	32 (15-75)	29	Partial liquid ventilation with perflubron	Conventional mechanical ventilation	Inhaled	day of admission	4 days	hospital discharge
de Felippe et al, 1993 [30]	41	35 (16-76)	n.r.*	Glucan	placebo	i.v.	12-145 hr (mean 46.2 hr) after admission	3-17 days	hospital discharge
Douzinas et al, 2000 [32]	39	32	24 (16-50)	Immunoglobulin	placebo (albumin)	i.v.	12 hr after admission	6 days	hospital discharge
Dries et al, 1998 [18]	73	31	34 (21-59)	rhIFN-γ	placebo	s.c.	within 30 hr of injury	21 days or hospital discharge	60 days
Glinz et al, 1985 [20]	150	39 (15-78)	30 (9-66)	Immunoglobulin	placebo (albumin)	i.v.	within 24 hr of starting mechanical ventilation	12 days	42 days
Livingston et al, 1994 [31]	98	30 (>16)	30 (±8)	rhIFN-γ	placebo	s.c.	day of admission	10 days	30 days
Marzi et al, 1993 [25]	24	32 (18-57)	34 (27-57)	superoxide dismutase	placebo (sucrose)	i.v.	within 48 hr of injury	5 days	14 days
Miller & Lim, 1985 [14]	28	n.r.	>10	Dextran + standard treatment	standard treatment	i.v.	within 12 hr of admission	5 days	4 weeks
Nakos et al, 2002 [26]	21	49 (35-67)	41 (24-62)	rhIFN-γ	placebo	inhaled	2nd or 3rd day after admission	7 days	hospital discharge
Nathens et al, 2006 [21]	268	42 (>17)	24 (±11)	Leukoreduced (<5 × 10^6 WBC) RBC transfusion	Nonleukoreduced (5 × 10^9WBC) RBC transfusion	i.v.	within 24 hr of injury	28 days	28 days
Polk et al, 1992 [22]	193	32 (>15)	33 (>20)	rhIFN-γ	placebo	s.c.	day of admission	10 days	90 days
Rhee et al, 2000 [23]	116	40 (>18)	20 (±11)	rhMAbCD18	placebo	i.v.	day of admission	single dose	hospital discharge
Rizoli et al, 2006 [27]	24	48 (>16)	26 (±11)	Hypertonic saline + Dextran	placebo (saline)	i.v.	upon arrival in de emergency department	single dose	hospital discharge
Seekamp et al, 2004 [16]	84	36 (17-72)	32 (17-59)	Anti-L-Selectin (Aselizumab)	placebo	i.v.	within 6 hr of injury	single dose	42 days
Vassar et al, 1991 [15]	48	36	31 (±3)	Prostaglandin E1	placebo	i.v.	24-48 hr after hospital admission	7 days	hospital discharge
Waydhas et al, 1998 [28]	40	33 (18-70)	41 (±13)	Antithrombin III	placebo (albumin)	i.v.	within 6 hr of injury	4 days	hospital discharge

IFN, interferon; ISS, injury severity score; i.v., intravenous; n, number; n.r., not reported; RBC, red blood cell; s.c., subcutaneous; WBC, white blood cell; * Trauma score 10, denoted as 'severe multiple trauma'.

### Outcomes

Among the outcome variables, most of the significant differences between the test and control groups were in inflammatory parameters, suggesting attenuation of SIRS, CARS, or both (Table 3). Only monoclonal antibodies against CD18 [23] exacerbated SIRS and hypertonic saline with dextran had a mixed effect on CARS [27]. Significant changes in infection and mortality rates were only measured in the studies testing IFN-γ [18,26], immunoglobulin [20,32], and glucan [29,30]. These were not the most recently published or largest studies, nor the studies with the longest follow-up, and did not differ from the other studies regarding the ages or ISS of the patients. Besides the test intervention, only the duration of the test intervention distinguished the studies that reported a significant efficacy in preventing adverse clinical outcome from those that did not; none of the single-dose interventions proved efficacious [16,17,19,23,27].

**Table 3:** Study results

			Infection		MOF, Mortality		Inflammation	
					
Test intervention		Study	Test group (relative to control)	Effect	Test group (relative to control)	Effect	Test group (relative to control)	Effect
Reduce immune paralysis	Plasma expander	Miller & Lim, 1985 [14]			Mortality 0 vs 0 n.s.	No effect	immune reactive capacity n.s.	No effect
		Rizoli et al, 2006 [27]	pneumonia 0.5% vs 0.5% n.s.	No effect	Mortality 0 vs 14.3% n.s., MOF score 1.68 vs 1.9 n.s.	No effect	WBC n.s.; decreased toward normal: CD11b, CD62L, CD16, and TNFα; increased toward normal: CD14, IL-1RA, and IL-10 all *P *< 0.05	SIRS↓ and CARS↓↑
		Bulger et al, 2008 [19]	nosocomial infections 18.2% vs 15.2% n.s.	No effect	ARDS-free survival, MOF, mortality 29.1% vs 22.2% n.s.	No effect		
	
	Immuno-globulin	Glinz et al, 1985 [20]	any 47% vs 68% *P *= 0.02, pneumonia 37% vs 58% *P *= 0.01, sepsis 18% vs 26% n.s.	↓	Mortality from infection* 12% vs 11% n.s.	No effect	acute phase proteins n.s.	No effect
		Douzinas et al, 2000 [32]	pneumonia 10% vs 61% *P *= 0.003	↓	Mortality rom infection* 0 vs 0	No effect	C3 and CH50 n.s., C4 increased p = 0.04, increased serum bactericidal activity *P *< 0.000001	CARS↓
	
	IFN- γ	Polk et al, 1992 [22]	major 39% vs 35%, minor 20% vs 28%, pneumonia 27% vs 24% n.s.	No effect	Mortality 9.2% vs 12.5% n.s.	No effect	HLA-DR increased *P *= 0.0001	CARS↓
		Livingston et al, 1994 [31]	major infection 48% vs 31% n.s.	No effect			WBC decreased *P *< 0.05, HLA-DR increased *P *< 0.05	SIRS↓ and CARS↓
		Dries et al, 1998 [18]	major infection 49% vs 58% n.s.	No effect	Mortality 13% vs 42% *P *= 0.017	↓	TNFα, IL-1β, IL-2, IL-4, IL-6 n.s.	No effect
		Nakos et al, 2002 [26]	ventilator-associated pneumonia 9% vs 50% p < 0.05	↓	Mortality 27% vs 40% n.s.	No effect	HLA-DR expression, IL-1β, phospholipase A2 all increased*P *< 0.05; total cells in BAL and IL-10 decreased *P *< 0.01	SIRS↓ and CARS↓
	
	Glucan	Browder et al, 1990 [29]	sepsis 9.5% vs 49% *P *< 0.05	↓	Mortality from sepsis* 0 vs 18% n.s.	No effect	IL-1β decreased *P *< 0.05, TNFα n.s.	SIRS↓
		de Felippe et al, 1993 [30]	pneumonia 9.5% vs 55% *P *< 0.01, sepsis 9.9% vs 35% *P *< 0.05, either or both 14.3% vs 65% *P *< 0.001	↓	Mortality: general 23.5% vs 42.1%, related to infection 4.8% vs 30% *P *< 0.05	↓		

Reduce hyper inflammation	Superoxide dismutase	Marzi et al, 1993 [25]			Mortality 17% vs 8.3% n.s. MOF score n.s.	No effect	WBC count, CRP, PMN-elastase and IL-6 n.s.; phospholipase A2 and conjugated dienes decreased *P *< 0.05	SIRS↓
	Antithrombin III	Waydhas et al, 1998 [28]			Mortality 15% vs 5%, MOF 20% vs 30% n.s	No effect	soluble TNF receptor II, neutrophil elastase, IL-RA, IL-6, and IL-8 n.s.	No effect
	Anti-CD18	Rhee et al, 2000 [23]	major and minor 38% vs 40% n.s.	No effect	Mortality 5.8% vs 6.7%, MOF score n.s.	No effect	WBC increased *P*-value not reported	SIRS↑
	Anti-L-Selectin	Seekamp et al, 2004 [16]	67% vs 55% n.s.	No effect	MOF n.s., mortality 11% vs 25% n.s.	No effect	WBC, IL-6, IL-10, neutrophil elastase, C3a, procalcitonin n.s.	No effect
	Leukoreduced blood	Nathens et al, 2006 [21]	30% vs 36% n.s.	No effect	Mortality 19% vs 15% n.s. MOF score 6.6 vs 5.9 n.s.	No effect		
	Perflubron	Croce et al, 1998 [24]	pneumonia 50% vs 3 75% n.s.	No effect	Mortality 8.3% vs 25% n.s.	No effect	WBC, neutrophils, IL-6, and IL-10 all decreased p < 0.01; capillary leak (BAL protein), TNFα, IL-1β, and IL-8 n.s.	SIRS↓
	Prostaglandin E1	Vassar et al, 1991 [15]	sepsis 28% vs 30%, major wound inf. 65% vs 72%, n.s.	No effect	Mortality 26% vs 28%, ARDS 13% vs 32%, MOF 30% vs 32% n.s.	No effect	PMN superoxide production increased toward normal *P *< 0.02	CARS↓

ARDS, acute respiratory distress syndrome; CARS, compensatory anti-inflammatory response syndrome; CRP, C-reactive protein; HLA, human leukocyte antigen; IL, interleukin; MOF, multiple organ failure; n.s., not significant; PMN, polymorphnuclear; SIRS, systemic inflammatory response syndrome; TNF, tumor necrosis factor; *, excluding deaths from cardiac arrhythmias secondary to a pulmonary embolus and myocardial infarction, intracranial pressure, and tracheostomy.

## Discussion

Although posttraumatic immune deregulation is apparent, the solution is not. In this systematic review we show that administration of immunomodulative interventions often leads to beneficial changes in the inflammatory response. Only administration of immunoglobulin, IFN-γ, or glucan was efficacious in reducing infection and/or mortality rate.

Immunoglobulin and IFN-γ both increase the antigen-presenting capacity of the host. After injury, circulating IgG levels are decreased [32]. Administration of exogenous immunoglobulins results in normalization of IgG concentrations and thus increases IgG-mediated antigen presentation. IgG is a plasma product obtained from healthy donors. IgG was given in the mentioned studies at a dose of 0.25 to 1.0 g/kg intravenously and reduced infections in trauma patients, which was more clearly seen in combination with antibiotics [20,32]. IFN-γ increases antigen presentation to lymphocytes via induction of HLA-DR expression on monocytes. Recombinant IFN-γ was given daily at a dose of 100 μg subcutaneously [18,22,26,31], but only had an positive effect on mortality [18] and infection [26] in two of four studies. Glucan, a component of the inner cell wall of *Saccharomycces cerevisiae*, reduces the immune paralysis via a different manner. It decreases prostaglandin release by macrophages but also stimulates bone marrow proliferation [29]. This bone marrow proliferation may be in favor in the late immune paralysis. Glucan was given at a dose of 50 mg/m^2 ^daily [29] or 30 mg every 12 hours [30], resulting in a reduced infection and mortality rate. All these seemingly effective interventions started on the day of admission and were continued until at least three to seven days after trauma.

As every systematic review, this study has its restrictions. A clear limitation of the trials is their relatively small sample size and the heterogeneity of interventions and study populations. Furthermore, we can not completely rule out publication bias. Yet, none of the studies report financial support by a pharmaceutical company and some studies show a negative result. Also, no other studies with immunoglobulin, IFN-γ, or glucan in trauma patients were found searching the clinical trial register database [33].

Challenges unique to the trauma population impede designing large RCTs. Polk et al. [22] note that patient homogeneity is difficult to achieve in multicenter trials because different centers tend to receive different patients. In addition, in the rush of the emergency care of severely injured patients, informed consent must wait until a family member is contacted [23] whereas the initiation of treatment cannot wait. Bulger et al. [19], Nathens et al. [21], and Rizoli et al. [27] solved this problem by gaining permission from their ethics committees to delay informed consent until after the initial treatment, but this approach is not always accepted. Furthermore, assessing patient eligibility for inclusion in the trial is time consuming. Delay to randomize patients can be avoided by using simple inclusion criteria. Nathens et al. [21] used only one criterion, the request of the physician for red blood cells for an expected transfusion, but were then faced with the possible dilution of treatment effect when they performed an intention-to-treat analysis because many randomized patients never received any blood products.

Based on the selected studies, general conclusions regarding the efficacy of potentially immunomodulative interventions cannot be drawn. As explained in the results section, the intended effects of the interventions on the inflammatory response differed. Furthermore, data from pilot studies [14,24-27] and phase II trials [16,23,24] should be used to steer future investigations rather than to draw definitive conclusions. Interventions that did not have a significant effect on clinical outcome may need to be administered earlier [25], continued longer [16,22,25,28], or need sequential specific timing to be effective [22]. Seekamp et al. [16] and Rhee et al. [23] explicitly chose for a single dose of an anti-inflammatory cytokine because they wanted to taper the initial hyper-inflammatory response without compounding the later immune paralysis. Timing is essential in accurate modulation of the immune response after trauma. The lack of a positive effect can be the result of wrong timing rather that to the drug itself. Consequently differences in timing between interventional drugs studied in this systematic review may contribute to disparity in outcome.

Besides changing timing, some authors recommended the use of larger doses [19,28]. Waydhas et al. [28] suggest that concomitant heparinization interfered with the immunomodulative effect of antithrombin III. The use of these drugs is inevitable in severely injured patients. Where theoretically promising approaches did not produce the results hoped for, sufficiently powered phase IV trails are needed.

Another impediment for drawing general conclusions is the fact that study populations differed greatly across the studies. For example, although Croce et al. [24] excluded patients with injuries thought to be lethal within 30 days of injury, others only excluded patients when the injuries were thought to be lethal within only one [28], two [16,20,21,23], or five [30] days. Similarly, de Felippe et al. [30] only included patients with concomitant head injury, whereas other researchers excluded patients with major head injury [16,19,23,28] or any head injury [14,29]. Mortality by severe head injury or massive bleeding may mask the effect of the interventional drug in an intention-to-treat trial, especially in trials with a small sample size.

Some researchers chose to exclude patients receiving steroids [24,25,31,32], because the efficacy of immunomodulative interventions is likely to be affected by simultaneous administration of steroids and/or antibiotics during care-as-usual [32]. However, this approach leads to a selection bias including patients that are more likely to have a favorable outcome.

Patient selection is imperative. Where no significant benefit was found for the test group as a whole, study authors postulated more specific inclusion criteria were necessary for future studies. For example, older patients [19,24,26], those with more severe injuries [19,23,26], patients needing 10 or more units of packed red blood cells [24], and those who had a longer time from injury to enrollment in the study [24] were more susceptible to organ dysfunction and thus likely to benefit more from immunomodulative intervention. Selection of patients at risk may favor the outcome where no significant difference was found in a broader group of patients. Researchers suggest future study participants be select based not only the injury severity, but also on sepsis [28] or inflammatory parameters [16] as Nakos et al. [26] did when they only randomized patients after ascertaining immune paralysis by measuring the HLA-DR in bronchoalveolar lavage.

Interpretations of the efficacy of immune modulating therapies in trauma patients remains difficult. More studies with similar study populations will aid comparison of the effect of different interventions in trauma patients.

## Conclusions

An array of potentially immunomodulative interventions have been tested in a heterogeneous group of trauma patients in level 1b and 2b RCTs. Reported changes in inflammatory parameters could indicate an attenuation of SIRS and/or CARS; however, they were not consistently accompanied by significant changes in infection and mortality rates. Administation of immunoglobulin, IFN-γ, and glucan was efficacious whereas none of the single-dose interventions were. Further trials powered to measure efficacy may reveal which immunomodulative interventions should be routinely implemented to save lives of trauma patients.

## Key messages

• Inflammatory complications, such as MOF and severe infection, are the most common cause of late death in trauma patients.

• An array of potentially immunomodulative interventions have been tested in a heterogeneous group of trauma patients in RCTs.

• Extensive disparity in study populations impairs inter-trial evaluation of efficacy of different (immunomodulative) interventions. Therefore, more standardized inclusion criteria are recommended.

• In most studies, the inflammatory parameters differed significantly between the test and control groups. However, significant changes in infection, MOF, and mortality rates were only measured in studies testing immunoglobulin, IFN-γ, and glucan.

• A recommendation can be made to administer immunoglobulin, IFN-γ or glucan to improve the outcome of trauma patients.

## Abbreviations

CARS: compensatory anti-inflammatory response syndrome; HLA: human leukocyte antigen; IFN: interferon; IL: interleukin; ISS: injury severity score; MARS: mixed antagonistic response syndrome; MOF: multiple organ failure; RCT: randomized controlled trial; SIRS: systemic inflammatory response syndrome; TNF: tumor necrosis factor.

## Competing interests

The authors declare that they have no competing interests.

## Authors' contributions

NS and LL conceived of and designed the study. NS and TV were involved in data acquisition, analysis, and interpretation and drafted the manuscript. LL and TV critically revised the manuscript for important intellectual content. All authors read and approved the final manuscript.

## Authors' information

NS and TV are PhD students at the Department of Surgery. LL is the Department's Professor of Traumatology.
